# Accuracy of New Deep Learning Model-Based Segmentation and Key-Point Multi-Detection Method for Ultrasonographic Developmental Dysplasia of the Hip (DDH) Screening

**DOI:** 10.3390/diagnostics11071174

**Published:** 2021-06-28

**Authors:** Si-Wook Lee, Hee-Uk Ye, Kyung-Jae Lee, Woo-Young Jang, Jong-Ha Lee, Seok-Min Hwang, Yu-Ran Heo

**Affiliations:** 1 Department of Orthopedic Surgery, Dongsan Medical Center, School of Medicine, Keimyung University, Daegu 42601, Korea; yhu4032@gmail.com (H.-U.Y.); oslee@dsmc.or.kr (K.-J.L.); 2 Department of Orthopedic Surgery, Korea University Anam Hospital, 73, Goryeodae-ro, Seongbuk-gu, Seoul 02841, Korea; opmanse@gmail.com; 3 Department of Biomedical Engineering, Keimyung University, Daegu 42601, Korea; segeberg@gmail.com (J.-H.L.); meen2378@naver.com (S.-M.H.); 4 Department of Anatomy, Dongsan Medical Center, School of Medicine, Keimyung University, Daegu 42601, Korea; chzh5240@naver.com

**Keywords:** developmental dysplasia of the hip, screening test, deep learning, Mask R-CNN

## Abstract

Hip joint ultrasonographic (US) imaging is the golden standard for developmental dysplasia of the hip (DDH) screening. However, the effectiveness of this technique is subject to interoperator and intraobserver variability. Thus, a multi-detection deep learning artificial intelligence (AI)-based computer-aided diagnosis (CAD) system was developed and evaluated. The deep learning model used a two-stage training process to segment the four key anatomical structures and extract their respective key points. In addition, the check angle of the ilium body balancing level was set to evaluate the system’s cognitive ability. Hence, only images with visible key anatomical points and a check angle within ±5° were used in the analysis. Of the original 921 images, 320 (34.7%) were deemed appropriate for screening by both the system and human observer. Moderate agreement (80.9%) was seen in the check angles of the appropriate group (Cohen’s κ = 0.525). Similarly, there was excellent agreement in the intraclass correlation coefficient (ICC) value between the measurers of the alpha angle (ICC = 0.764) and a good agreement in beta angle (ICC = 0.743). The developed system performed similarly to experienced medical experts; thus, it could further aid the effectiveness and speed of DDH diagnosis.

## 1. Introduction

Developmental dysplasia of the hip (DDH) is a hip joint disease with various possible causes, including genetics, intrauterine factors, and cultural practices. Although the incidence rates vary by race and ethnicity, many developed countries report DDH rates ranging from 1.5 to 20 cases per 1000 births [1,2]. DDH causes multiple spectrum disorders, such as mild capsular laxity, acetabular deficiency, subluxation, and hip dislocation. This spectrum gradually progresses in severity, with treatment becoming more difficult as the patient ages and grows. Fortunately, DDH can be treated simply with a brace or splint when diagnosed early [3,4]. Otherwise, secondary anatomical changes caused by delayed diagnosis could require surgical treatment [5,6]. If the diagnosis is further delayed or DDH is not diagnosed, osteoarthritis of the hip joint may occur at an early age, requiring major surgical treatment (e.g., hip replacement) [7]. Therefore, DDH prognosis depends on early diagnosis and treatment. Recently, many countries (e.g., Germany, Israel, Korea) have conducted DDH screening tests on newborns at about six weeks of age because of the advantages of early diagnosis [8,9,10].

DDH diagnosis is done through radiography, physical examination, and US imaging. Radiography is an effective DDH diagnostic method when femoral head ossification occurs in children aged more than six months. Similarly, physical examinations can only diagnose complete hip dislocation. Thus, neither are adequate early DDH screening methods. The preferred means of early DDH screening is ultrasonographic (US) imaging because it can capture cartilage conditions before femoral head ossification occurs. In addition, it can be used to diagnose mild spectrums of DDH aside from dislocation. Moreover, there is no concern about ionizing radiation by US imaging.

**Figure 1:**
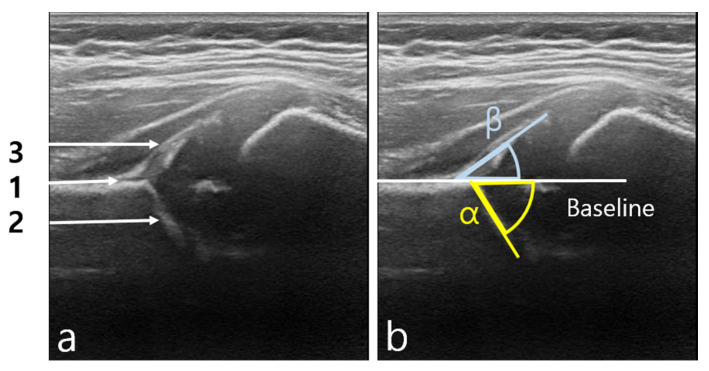
(**a**) A standard hip ultrasonographic (US) image should show three key anatomical structures: 1. vertical ilium, 2. lower margin of Os ilium, and 3. labrum. (**b**) Alpha (α) and beta (β) Graf angles in a US image of the hip.

The most common DDH US analysis is the Graf method [11], which measures a bony acetabular depth or coverage of cartilaginous with labrum in a coronal US image of the hip (Figure 1). However, this method has high interoperator variability [12,13,14] that could lead to misdiagnosis of DDH in half of the infant screenings and up to three-quarters in neonatal screenings [13]. The primary sources of image acquisition variability between operators arise from the manual selection of anatomical points for calculating angles (i.e., tri-radiate cartilage, the acetabular bone edge, the lower edge of the iliac bone, and the end of the acetabular labrum). An artificial intelligence (AI)-based computer-aided diagnosis (CAD) system can standardize image acquisition and reduce diagnosis time, thus improving the accuracy and objectivity of DDH diagnosis.

The concept of CAD emerged in the 1970s when scanned medical images were analyzed using a computer. In 1998, LeCun et al. [15] laid the foundations for today’s deep learning by outlining the convolutional neural network (CNN) framework—a neural network being a derivative of mathematical models that simulate the structure and function of biological neural networks. As graphics processing unit (GPU) images improved with advances in technology, deep learning, mainly through CNNs, has become a conventional approach to medical imaging and is even used for genetic analysis [16,17,18]. CNN is an effective tool in image recognition, especially in judging image borders and colors. In addition, it has shown excellent diagnostic performance based on endoscopic images and magnetic resonance imaging (MRI) [19,20]. Hence, multiple studies on DDH diagnosis using US images of the hip joint have been conducted [21,22].

Region-based CNNs (R-CNN) apply CNN for object detection in an image. Object detection consists of proposing a region in an image where an object is supposed to exist and analyzing the region. Despite R-CNN’s excellent object detection accuracy, its processing is slow because it performs CNN for too many regions in an image proposed by its selective search feature. Thus, Fast R-CNNs have been proposed to improve efficiency [23]. This type of R-CNN performs CNN for the entire image, proposes regions of interest (RoIs), and then uses the region of interest pooling (RoIPool) to make the objects of interest a feature of a fixed resolution. However, Fast R-CNN’s process is still time-consuming. Faster R-CNNs hasten the process using a region proposal network (RPN) to propose a region from a feature map. Mask R-CNN is based on Faster R-CNN and has the advantage of automatic image segmentation. Specifically, it can define and propose RoIs based on boundary contours of the anatomical structure in hip US images using the region of interest alignment (RoIAlign) rather than merely using boxes. Furthermore, it also can find specific points in an image [24].

Because US images are generally of low resolution and in grayscale, US imaging is at a disadvantage. Specifically, a large amount of image data are required to construct an artificial intelligence (AI) system that automatically detects anatomical structures and obtains the needed angles without human intervention. This disadvantage obstructs research and development in a single institution. Thus, this study segmented a relatively small number of US images using Mask R-CNN and developed a two-stage multi-detection method AI system in a single research institution to extract the needed data from the segmented images. To the best of the researchers’ knowledge, this is the first study to estimate automatically detected key points in US images using a deep neural network model.

## 2. Materials and Methods

The institutional review board approved the study (IRB No. DSMC 2019-10-003). The study’s dataset consisted of 1243 hip US images from 168 infants, using a 12.5 MHz linear probe with HD15 and HD7 XE ultrasound systems (Philips, Bothell, WA, USA) during a DDH neonatal screening and diagnosis program between 2002 and 2019, retrospectively. Of the total 1243 images, 289 images were randomly selected for AI training, 33 for validation, and the remaining 921 for testing.

### 2.1. Images

One radiologist and one pediatric orthopedic surgeon collected all images, both with more than 10 years of US DDH screening experience. These images were anonymized and documented in the vertically oriented standard plane as Digital Imaging and Communications in Medicine (DICOM) files.

Three lines were drawn to distinguish the localized anatomical structures that represent the alpha and beta Graf angles. However, alpha and beta angle measurements without parallelization to the vertical ilium are ineffective when evaluating hip dysplasia. Thus, a “check angle” was used to define the angle created by the iliac wing and baseline. Check angle values measured within ± 5° by both a pediatric orthopedic surgeon (referred to in this study as “doctor”) and the AI system were classified as the “appropriate image”. These were the only alpha and beta angles used to evaluate AI performance.

### 2.2. Multi-Detection Type Artificial Intelligence (AI)

**Figure 2:**
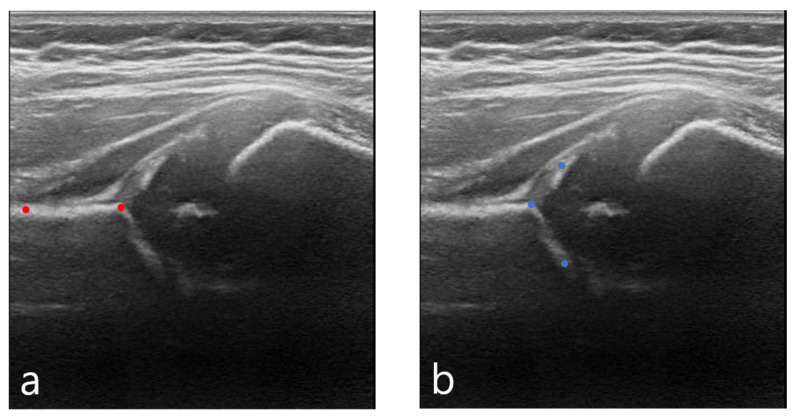
Calculating Graf angles using four points: (**a**) Two “check angle” points determine the appropriateness of the image. (**b**) Three points are used to calculate the alpha and beta angles, where one point on the tri-radiate cartilage overlaps with a check angle point.

In previous studies, the ilium, the acetabular roof, and the labrum in ultrasound images were segmented. Furthermore, the angles created by these three structures were calculated through an algorithm to obtain the alpha and beta Graf angles [21,22]. However, in practice, doctors calculate the same angles by extracting measurement points based on anatomical understanding. In this study, the ilium’s, the acetabulum’s, and labrum’s shapes were segmented using the Mask R-CNN method. Then, two check angle points and another three points were determined for calculating the alpha and beta angles. Of these points, one is an overlap of a check angle point and the middle tri-radiate cartilage point. Overall, four points were used to calculate the alpha and beta angles, as seen in Figure 2.

#### 2.2.1. Segmentation

**Figure 3:**
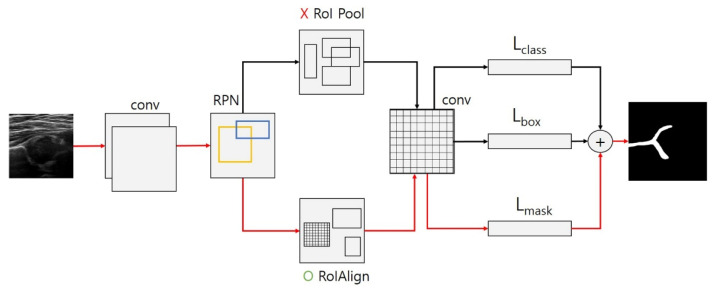
Comparing the Mask region-based convolutional neural network (Mask R-CNN; red arrows) and Faster R-CNN (black arrows) methods for image segmentation.

Segmentation was done using the Mask R-CNN technique, wherein a mask head is added to a Faster R-CNN [24]. Specifically, the Mask R-CNN adds a mask branch to determine whether a particular pixel corresponds to an object. Mask R-CNN is similar to Faster R-CNN, using region proposal network (RPN) to extract features and classify and tighten bounding boxes. Faster R-CNN uses RoIPool as a feature extraction method for quantifying each RoI region, thus solving the problem of differing RoI feature sizes at different scales by max pooling. However, this process causes spatial information loss, leading to the displacement of the original image RoI and extraction features. To solve this problem, Mask R-CNN replaces the RoI pooling of Faster R-CNN with RoI alignment (RoIAlign) to mark the object area. Figure 3 illustrates the image segmentation process of both Mask R-CNN and Faster R-CNN methods. The red arrows depict the Mask R-CNN method using region of interest alignment (RoIAlign) to draw a precise bounding box that matches an object and extracts specific key points from the object of interest. In this case, the areas of the ilium, acetabulum, and labrum were extracted using a mask head. The region of interest alignment (RoIAlign) and feature pyramid network structures were then used to extract features for the residual neural network (ResNet) algorithm consisting of 50 layers modified with the background Mask R-CNN algorithm. The black arrows show the Faster R-CNN method using the region of interest pooling (RoIPool), which creates a square-bound box containing an object.

#### 2.2.2. File Conversion

Each patient’s data, including his/her age and sex, were documented as a DICOM file. All identifying information was removed, following ethical guidelines. The anonymized data were then converted into 256-bit grayscale portable network graphics (PNG) image files.

#### 2.2.3. Layer Extraction

**Figure 4:**
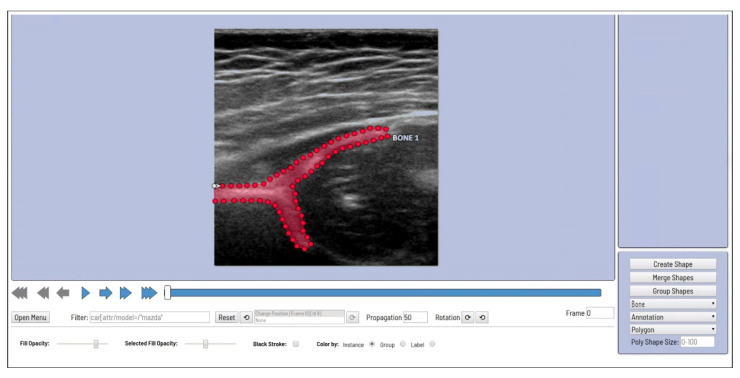
Sample model training using Computer Vision Annotation Tool (CVAT).

AI training started with extract image segmentation of the US images. The Computer Vision Annotation Tool (CVAT; Massachusetts Institute of Technology, MA, USA), a free online program that helps annotate videos and images for computer vision algorithms [25], was used to designate the area for the corresponding mask position (Figure 4).

#### 2.2.4. First Training

**Figure 5:**
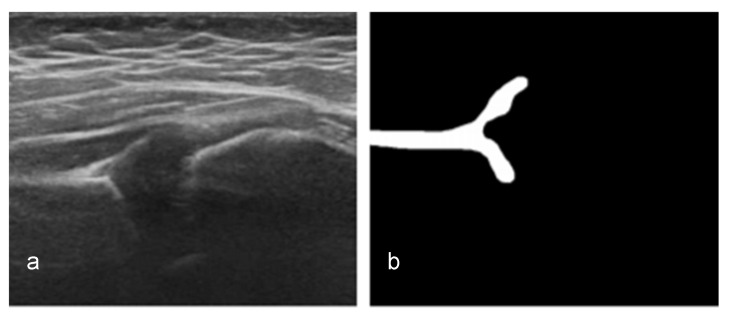
(**a**) Original image ultrasonographic image of the hip. (**b**) The same image after the initial conversion.

Using CVAT’s point tool, the key anatomical structures in the US images were designated, while the extensible markup language (XML) data were obtained using the extraction tool. The first AI training used the initial modeling data composed of the generated XML file and image file. In this stage, the areas corresponding to the ilium, acetabulum, and labrum were converted to white (RGB 255, 255, 255) and the rest to black (RGB 0, 0, 0) as seen in Figure 5.

#### 2.2.5. Second Training

**Figure 6:**
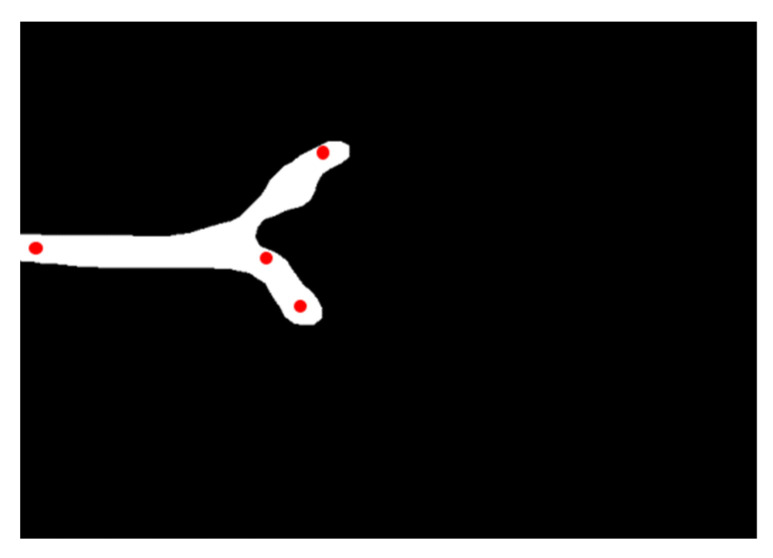
Point marks for the second training based on the first training’s resulting image.

Using CVAT, the points corresponding to the tri-radiate cartilage, the acetabular bone edge, the lower edge of the iliac bone, and the end of the acetabular labrum were marked with red dots. The secondary training was performed using the resulting image data of the first training and an XML file marked with the four key points (Figure 6).

#### 2.2.6. Final Result

**Figure 7:**
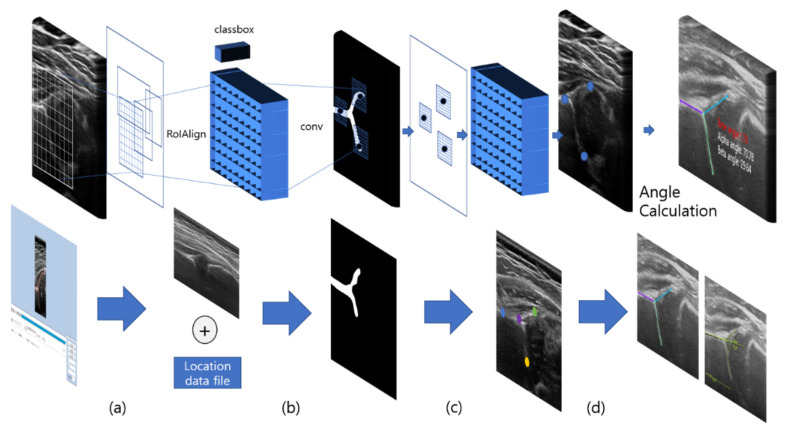
Diagrammatic illustration of the multi-detection type artificial intelligence (AI) training courses: (**a**) area learning training using CVAT Program, (**b**) initial image file conversion, (**c**) the secondary point-marking training, and (**d**) the final result of a multi-detection-trained AI system.

The multi-detection process was completed after the second training, wherein the alpha and beta angles were obtained. A diagram of the implementation process is shown in Figure 7.

### 2.3. Network Training

The training takes 40,000 steps in total, with validation at every 1000 steps to verify the best model selection. The learning rate and weight decay were 0.01 and 0.0001, respectively, while the stochastic gradient descent used a batch size of 10. The following data augmentation settings were applied: up/down-left/right random shift, −5–5%; random scale, 80–100%; random rotation, −15–15°; and random brightness, 75–100%. In addition, the Random Gaussian Noise setting was used for the robustness of various ultrasonic noises and brightness.

### 2.4. Statistical Analysis

The check angle and Graf angles of the same images calculated by a pediatric orthopedic surgeon and the AI were compared. The R language version 3.3.3 (R Foundation for Statistical Computing, Vienna, Austria) program was used for all statistical analyses. Percent agreement, positive percent agreement, and Cohen’s kappa were computed to assess inter-rater reliability (IRR) of the AI’s and doctor’s determined check angles. Furthermore, the intraclass correlation coefficient (ICC) was measured to evaluate the agreement of the AI’s and the doctor’s computed alpha and beta angles.

## 3. Results

### 3.1. Criteria for Data Classification

Before the performance evaluation, finding an image capable of measuring the alpha and beta angles from the 921 test data images was necessary. Two criteria were set: (1) all four key anatomical structures and their respective points (i.e., tri-radiate cartilage, the acetabular bone edge, the lower edge of the iliac bone, and the end of the acetabular labrum) were visible; and (2) the iliac wing is parallel to the baseline.

#### 3.1.1. Data Classification Based on First Criteria

Hence, two measurers categorized the images based on detectability and as “detectable” if the key anatomical structures and their respective points are observable. The images were classified as “undetectable” if otherwise.

The doctor classified 542 images as “detectable” images; in contrast, the AI classified 555 images as “detectable” images. Overall, the 512 images were commonly categorized as “detectable” images. The 409 images that at least one doctor or AI could not detect were classified as “fail detection” (Table 1).

#### 3.1.2. Data Classification Based on Second Criteria

Images categorized as “detectable” in the first classification were classified once again according to whether the correct alpha and beta angles could be measured. The image was defined to measure the correct alpha and beta angles when the baseline and the iliac wing are generally parallel. Thus, if images of the iliac wing parallel to the baseline measured a check angle value within ±5°, they were classified as “OK check angle” and meet the specified criteria. Otherwise, they were classified as “error check angle”.

The 512 commonly categorized “detectable” images from the performance evaluation were used to classify images according to check angle. Of these images, 320 were classified as “OK check angle” by both the doctor and AI (Table 2). The interobserver check angle agreement shows that the percent agreement at 80.9% and kappa coefficient of 0.5 (95% confidence interval 0.52; 0.5) is reasonably good. In addition, a positive agreement of 0.87 existed between the observers.

### 3.2. Statistical Result of Graf Angle Measurement

**Figure 8:**
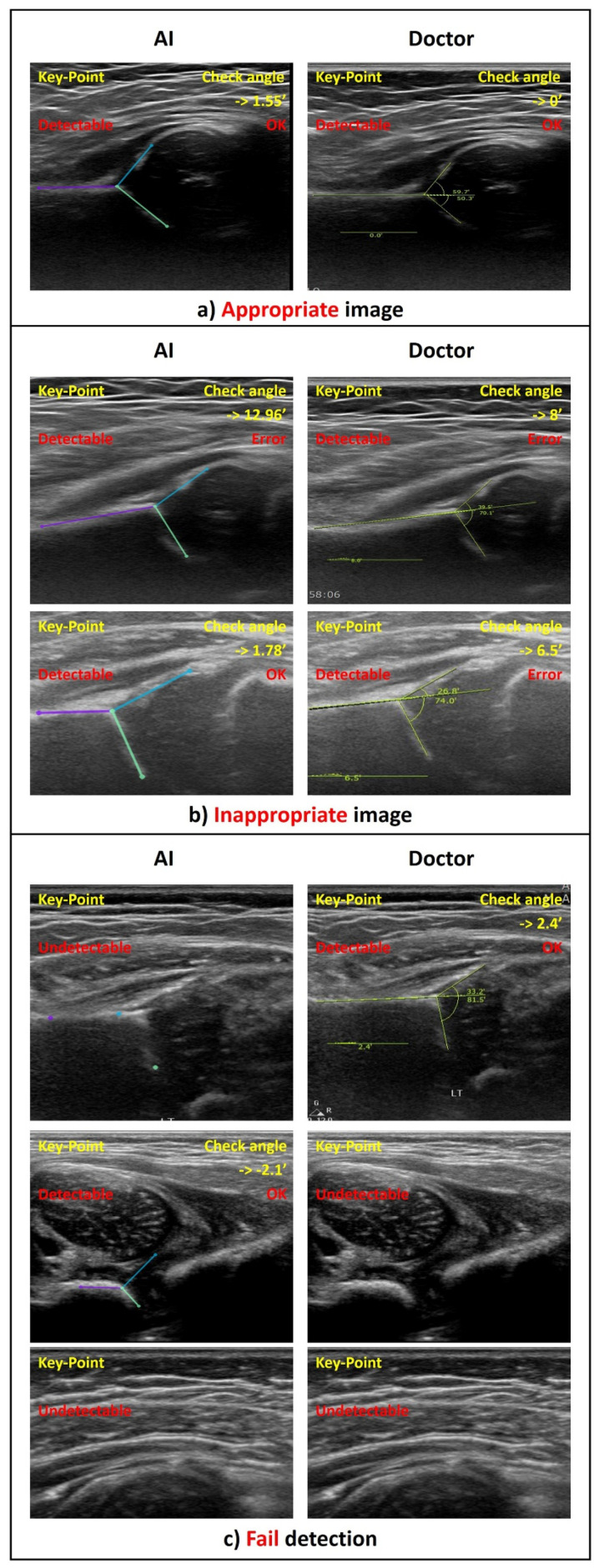
Examples of ultrasonographic image classifications in the study: (**a**) Case of the appropriate image where detection of all key points was possible, the measured check angle was less than 5° for both observers, and the alpha and beta angles were evaluated (*n* = 320). (**b**) Case of the inappropriate image where detection of all key points was possible, but the check angle was more than 5° for at least one observer (*n* = 192). (**c**) Case of the fail detection where at least one observer could not detect all key points (*n* = 409).

**Figure 9:**
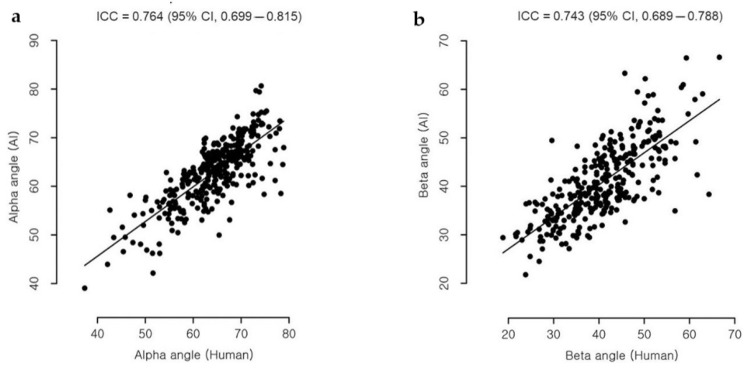
(**a**) Correlation scatter plot of the alpha angles measured by the artificial intelligence (AI) system and the doctor (Human). (**b**) Correlation scatter plot of the beta angles measured by the artificial intelligence (AI) system and the doctor (Human).

**Figure 10:**
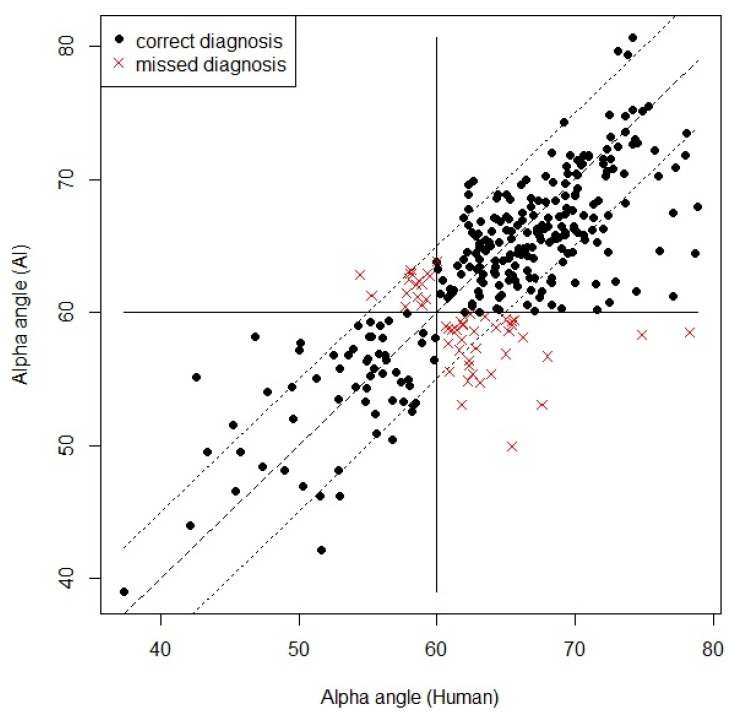
Scatter plot of the alpha angles measured by the artificial intelligence (AI) system and the doctor (Human) on the *y*-axis and *x*-axis, respectively. The black points between the dotted lines represent a correct diagnosis wherein the difference in the observers’ measurements is less than 5°. These specific points account for 74% of the correct diagnoses made. Furthermore, the black points on the first and third quadrants represent correct diagnoses. Following the Graf method, the observers had the same normal and abnormal classifications based on a 60° alpha angle. These specific points account for 84% of all the measurements made. In contrast, values expressed as red crosses represent the observers’ different normal and abnormal classification results. These crosses represent 16% of all the measurements made.

Only images classified as “detectable” with “OK check angle” (thus meeting both criteria) were defined as appropriate images and considered suitable for an appropriate DDH diagnosis. Figure 8 shows examples of the image classification. As a result, the alpha and beta Graf angles of the 320 appropriate images were compared. Similar to Cicchetti [26], the study considers an intraclass correlation coefficient (ICC) > 0.70 acceptable. The resulting ICC revealed excellent agreement for the alpha angle (ICC = 0.76) and good agreement for the beta angle (ICC = 0.74) (Table 3 and Figure 9). For the alpha-angle, 74.7% of images displayed a discrepancy of less than 5° between the observers. In addition, the observers agreed in the classification of 84.3% of cases as being normal or abnormal, as seen in Figure 10. The mean absolute deviation (MAD) of the alpha angle was 3.4 while it was 4.5 for the beta angle. The standard deviation of differences was 4.5°, similar to the interhuman observer variability reported in previous studies (Table 4) [12,27].

## 4. Discussion

US imaging is a noninvasive and safe diagnostic tool that offers excellent results for DDH screening [8], especially when using the Graf method. However, intraobserver and interobserver variances in the measurement of hip US imaging remain an issue [12,13,14]. The primary source of variability arises from the selection of anatomical points for calculating angles. In response, an automatic key point system for DDH diagnosis was developed based on a multi-detection deep learning system. Because it is a multi-training system, small data sizes can be analyzed.

The agreement between the AI system and the doctor was excellent in the alpha-angle (ICC = 0.76) and good for the beta-angle (ICC = 0.74) calculations. In addition, by analyzing 320 hip US images, the AI system differentiated normal and abnormal hip US images with a match rate of 84.37% to the doctor. These results show that the system improved from previous methods [12,28]. Simon et al. [28] evaluated concordance between orthopedic surgeons and pediatricians for the diagnosis of DDH, and the reported concordance rate was 82.91%, with class correlation coefficients for alpha angles (ICC = 0.72) and beta angles (ICC = 0.34). Roovers et al. [12] reported a match rate of 85% between doctors in the classification of 200 hip US images as normal or dysplasia. Thus, the proposed new deep learning system produced a higher interobserver agreement, and results with experienced orthopedic practitioners can be regarded as comparably good.

Our study had several limitations. First, although there were many images in the dataset, the number of images and image sources was still small. Thus, the results cannot be generalized. Second, the study sample of doctors was small, having two medical experts in data collection and one in measuring the Graf angles in images of the control group. Because of interoperator/interhuman variability, it is necessary to compare the images collected by more US imaging operators and angles calculated by more doctors. Finally, the study did not validate the data using external datasets.

DDH becomes challenging to treat when the diagnosis is delayed; hence, screening tests are essential. This new AI-based CAD system can be a helpful tool for inexperienced physicians to measure angles in hip images and lessen the interobserver variability of DDH diagnosis.

## 5. Conclusions

A CAD deep learning model based on segmentation and key-point multi-detection using Mask R-CNN was developed for DDH screening and diagnosing. The system’s performance was comparable to that of human experts. Hence, it can be used as an auxiliary method for DDH screening.

## Figures and Tables

**Table 1:** Data classification based on first criteria.

	Subgroup	*n* (%)
Doctor		921 (100)
	Detectable	542 (58.9)
	Undetectable	379 (41.1)
Artificial Intelligence		921 (100)
	Detectable	555 (60.3)
	Undetectable	366 (39.7)
Doctor and Artificial Intelligence		921 (100)
	Commonly detectable	512 (55.6)
	Fail detection	409 (44.4)

**Table 2:** Data classification according to the check angles of detectable images.

	*n* (%)	Check Angle (Doctor) OK	Check Angle (Doctor) Error
All detectable image	512 (100)		
Check angle (AI): OK		320 (86.7)	49 (34.3)
Check angle (AI): Error		49 (13.3)	94 (65.7)

**Table 3:** Intraclass correlation coefficient (ICC) scatter plot between artificial intelligence (AI) and doctor using alpha and beta angle as measurement.

	ICC	95% Confidence Interval	Agreement
Alpha angle	0.764	0.699–0.815	Excellent
Beta angle	0743	0.689–0.788	Good

**Table 4:** Mean and standard deviation of angles measured from AI and doctor.

	*n* (%)	Artificial Intelligence (AI)	Human	AI–Human	Mean Absolute Deviation (MAD)
Alpha angle	320 (100)	62.843 ± 6.514	64.117 ± 7.12	−1.274 ± 4.555	3.470
Beta angle	320 (100)	40.785 ± 7.69	40.679 ± 8.71	0.106 ± 5.9	4.501

## Data Availability

The data presented in this study are available on request from the corresponding author. The data are not publicly available due to patient’s right to privacy.
